# The Ins and Outs of Chemokine-Mediated Immune Cell Trafficking in Skin Cancer

**DOI:** 10.3389/fimmu.2019.00386

**Published:** 2019-03-07

**Authors:** Andrew O. Yam, Tatyana Chtanova

**Affiliations:** ^1^Immunology Division, Garvan Institute of Medical Research, Sydney, NSW, Australia; ^2^Faculty of Medicine, St Vincent's Clinical School, University of New South Wales, Sydney, NSW, Australia

**Keywords:** immune cell, chemokine, skin cancer, melanoma, migration

## Abstract

Recent studies of the patterns of chemokine-mediated immune cell recruitment into solid tumors have enhanced our understanding of the role played by various immune cell subsets both in amplifying and inhibiting tumor cell growth and spread. Here we discuss how the chemokine/chemokine receptor networks bring together immune cells within the microenvironment of skin tumors, particularly melanomas, including their effect on disease progression, prognosis and therapeutic options.

## Introduction

Chemokines and their receptors are an intrinsic part of immune cell trafficking. They orchestrate cell-to-cell interactions during normal immune responses and are fundamental for directing immune cells to sites of inflammation (1). Chemokines are chemoattractant molecules 8–10 kDa in size that can induce directed migration in nearby cells. They bind to G-protein coupled chemokine receptors and can be divided into four families based on the pattern of their cysteine residues: CC, CXC, CX3C, and C.

Chemokine receptor-ligand pairs play a key role in tumorigenesis in addition to infection and inflammation (2–4). Within the tumor microenvironment, both host and cancer cells can release a range of chemokines, leading to recruitment and activation of different immune cell subsets that can either enhance or inhibit anti-tumor immunity (5–9). These subsets include tumor infiltrating lymphocytes and cells of the innate immune system such as neutrophils, dendritic cells (DCs) and monocyte/macrophages.

Tumors can be broadly divided into T cell-inflamed and non-inflamed tumors (10). T cell-flamed tumors are characterized by type 1 interferon expression and chemokines that attract T cells and antigen presenting cells (10). T cell infiltration of tumors has been associated with improved survival and response to immunotherapy (11). However, in some cases infiltrating cytotoxic T cells can be functionally exhausted, whereas recruitment of regulatory T cells (Tregs) promotes tumor growth by suppressing anti-tumor responses (12). Innate immune cells including macrophages and neutrophils, which can have either pro- or anti-tumor functions, also contribute to the outcome of the tumor immune response (13–15). Thus, the potential exists to manipulate chemokine signaling in such a way as to enhance recruitment of cytotoxic cells into non-inflamed tumors or to shift the balance in inflamed tumors in favor of effector rather than regulatory cells.

In addition to their primary role as chemoattractants, chemokines are involved in other tumor-related processes and may be exploited by tumors to promote tumor cell growth (2), angiogenesis (16), and metastasis (17). A comprehensive discussion of the role of chemokines in cancer has been the subject of several recent reviews (18–20). Here we shall, therefore, focus on the part played by chemokines and their receptors in immune cell recruitment in skin cancers with specific emphasis on melanoma, since the vast majority of the available data on skin cancer comes from studies of patients with melanoma or murine models of melanoma.

## Chemokine–Driven Innate Immune Cell Infiltration in Skin Cancer

Innate immune cells, among them DCs, macrophages, neutrophils and NK cells form an important part of the tumor immune milieu. Their role in skin cancers can be 2-fold: either they can mediate direct anti-tumor toxicity through promoting cytotoxic T cell recruitment and activation or they can contribute to inhibition of anti-tumor responses through inhibitory interactions with other cells and secretion of suppressive molecules (Table 1).

**Table 1:** Chemokines implicated in regulating immune responses in melanoma.

**Chemokines**	**Cellular sources**	**Regulation**	**Effect on innate immunity**	**Effect on adaptive immunity**
CCL2	Stromal/Immune cells (21) Melanoma (22)	*Activation* TNF (23)	Low levels of CCL2: recruitment of macrophages and enhanced angiogenesis and tumor formation (23) High levels of CCL2: infiltration of anti-tumor macrophages leading to tumor regression (23)	Expression correlated with increased tumor CD3+, CD8+ T cell infiltration (6, 24, 25), improved response to checkpoint inhibitors (25) and survival (24)
				Lack of CCL2 or CCR2 reduced γδ T cell infiltration and increased tumor growth (26)
CCL3-4	Intratumoral myeloid derived suppressor cells (MDSCs) (8)			CCL3-4 in melanoma correlated with increased CD3+, CD8+ T cell infiltration (6, 24), and improved survival (24). Increased CCL4 in melanoma pre- and post- anti-CTLA4 (ipilimumab) treatment was associated with T cell infiltration and response to treatment (27)
CCL5		*Inhibition* BECN1 autophagy gene (28)	NK cell recruitment into melanoma, associated with tumor regression (28)	CCL5 synergized with CXCL9 to recruit T cells into melanoma (29)
CCL5, XCL1	NK cells (5)	*Inhibition* PGE2 (5)	DC recruitment into melanoma and tumor growth inhibition (5)	
CCL20	Tumor macrophages (30)	*Activation* TNF (30)	DC recruitment into melanoma and T cell-dependent inhibition of tumor growth (31)	
CCL21	Melanoma cell lines MDA-MB-435S (32) and B16F10 (9)			Increased Treg infiltration and tumor growth (9)
CCL22	Melanoma cell line B16F10 (33)			Overexpression of CCL22 in skin diverted Treg cells from lung metastasis leading to inhibition of metastatic growth in the lung (33)
CXCL1,2,5	Tumor neutrophils (7)	*Inhibition* IFN-β (7)	Neutrophil recruitment into melanoma leading to angiogenesis and tumor growth (7). CXCL5 promoted neutrophil dependent tumor cell migration into lymphatic vessels (34).	
CCL5, CXCL9 - 11	CCL5: Intratumoral MDSCs (8) CXCL9-10: CD103+ DC (5) CXCL9-11: Tumor endothelial cells (35)	CXCL9-10: *Activation* IFN (36) *Inhibition* Adenosine (37)		CCL5 and CXCL9-11 expression in melanoma correlated with increased CD8+ T cell infiltration (6), improved survival (24) and response to adoptive T cell therapy (38) CCL5, CXCL9-10 were associated with response to MAGE-A3 vaccine (39) CXCL9-11 recruited γδ T cells into melanoma (40)
CXCL12	Tumor macrophage (41) Tumor endothelium (42)		DC recruitment into melanoma, CD8+ T cell dependent tumor growth reduction (43)	CXCL12 recruited CTLs into melanoma (44)

### DCs

DCs are not only the professional APC responsible for activating naïve T cells in secondary lymphoid tissue (45), but can also influence cytotoxic T cell recruitment into tumors. Thus, CXCL9/10, a chemokine associated with CD8+ T cell infiltration (6, 46), was produced by Batf3-driven CD103+ DCs present in the melanoma microenvironment (47). Consistent with an important role for CD103+ DCs in trafficking antigen and T cell activation in tumor draining lymph nodes (45), depletion or lack of this subset prevented intrinsic and adoptive T cell recruitment into tumors (47). However, since Tregs also express CXCR3, the receptor for CXCL9/10 (48), this may also promote recruitment of immunosuppressive cells. Expression of the DC chemoattractant CCL20 led to DC recruitment and T cell-dependent inhibition of B16 murine syngeneic melanoma (31). Similarly, the positive association of CXCL12 with cytotoxic T cell recruitment was related to the presence of DCs within melanoma. Transfection of CXCL12 into B16 melanoma cells induced DC accumulation within the tumor and reduced tumor growth in a CD8+ T cell-dependent manner (43). Likewise, recruitment of conventional DCs into melanoma by CCL5 and XCL1, whose production was dependent on NK cells, promoted tumor growth control (5). Supporting this data, the combination of NK and DC tumor gene signatures from The Cancer Genome Atlas correlated with melanoma patient survival (5), while NK cells in melanoma predicted response to anti-PD1 by regulating the DC abundance in tumors through secretion of cytokine FLT3LG (49).

### Macrophages

Macrophages are also frequently found in solid tumors including melanomas where they may have dual roles leading to their classification into anti-tumor M1 and inhibitory M2 subtypes (14). M2 macrophages preferentially express pro-angiogenic factors and metalloproteinases, which contribute to a microenvironment conducive to tumor growth (14, 50). CCL20-producing tumor associated macrophages were associated with tumor progression and worse survival possibly because they co-expressed pro-tumor cytokine TNF and pro-angiogenic VEGF-A (30).

The macrophage chemoattractant CCL2 is expressed on melanoma cells (22) and its effect on macrophage function in melanoma is concentration-dependent (23). Low levels of CCL2 led to modest macrophage infiltration and tumor formation by promoting angiogenesis, whilst higher levels were associated with increased macrophage infiltration and tumor regression. Furthermore, expression of CCL2 in human IIB-MEL-J melanoma increased intra-tumor macrophage infiltration and tumor growth while macrophage-depleted mice developed smaller tumors (51). CCL2 macrophage recruitment into melanoma was associated with higher-grade melanoma (31) and promotion of tumor growth through increased TNF-α dependent vascularization (23, 51).

### Neutrophils

Neutrophils are the third member of the innate immune cell repertoire to play a vital role in skin cancer (52, 53). The full extent of neutrophil functions in skin cancers is yet to be revealed as several studies suggested that like macrophages, neutrophils can be tumor-promoting or anti-tumor (13, 15). Neutrophils migrate into melanoma using the CXCR2 chemokine receptor in response to its ligands CXCL1, CXCL2, and CXCL5 expressed in melanoma (7). CXCL5 was upregulated in human stage T4 melanoma biopsies, which correlated with greater neutrophil infiltration and locoregional metastasis, when compared to stage T1 human melanomas (34). Furthermore, in a metastatic murine xenograft model, overexpression of CXCL5 in human melanoma cells elicited increased neutrophil recruitment and neutrophil dependent tumor cell migration into lymphatic vessels leading to lymph node metastasis (34).

Melanoma grown in mice lacking CXCR2 display reduced inflammation, neutrophil recruitment and tumor growth (54). Notably IFN-β knockout mice had increased CXCL1 and CXCL2 expression, suggesting that IFN-β is likely to be an intrinsic regulator of neutrophil infiltration (7, 55). In another model of melanoma, CXCL6 was shown to be important for neutrophil infiltration into tumor. Moreover, anti-CXCL6 monoclonal antibodies reduced neutrophil recruitment, which had an effect of inhibiting melanoma growth (56).

### NK Cells

NK cells are innate lymphocytes with a crucial role in anti-viral and tumor defense (57–59). Their homing to melanomas, where they mediated growth regression, was dependent on CXCR3 as CXCR3-deficient NK cells failed to migrate into melanoma (60). Furthermore, higher levels of CCL5 in melanoma increased NK cell infiltration, reduced tumor size and could be used as a predictor for patient survival (28).

## Chemokine-Driven Effector T Cell Recruitment in Skin Cancer

T cells have crucial roles to play in controlling certain infections and cancers including those located in the skin. The presence of CD8+ T cells in melanomas (61–63) as well as in other cancers (64–66) is associated with better clinical outcomes. Furthermore, patients with advanced melanomas have benefited from therapies designed to increase T cell infiltration of tumors including checkpoint inhibitors, T cell modulating cytokines (IL-2, IFN-γ) and adoptive T cell transfer. Therefore, a thorough understanding of chemokine-mediated T cell trafficking in skin cancer (Table 1) is crucial for achieving better treatment outcomes.

One of the earliest studies to link chemokine expression in human melanoma to CD8+ T cell infiltration used gene expression arrays to identify CCL2-5 and CXCL9 as preferentially expressed in T cell rich patients' tumors (6, 29). Their corresponding chemokine receptors were upregulated on effector compared to naïve CD8+ T cells from normal controls (6). In another study higher expression of CCL3-5 and CXCL9-11 in human melanoma tissue was associated with increased CD8+ T cell recruitment and patient survival (24, 67). Furthermore, analysis of chemokines in melanoma prior to treatment identified CCL2 and CXCL9-12 as elevated in responding patients compared to non-responding patients (25). Tumor samples from patients with better clinical responses also had higher T cell counts around the invasive margins (25).

Expression of CCR5 and CXCR3 (receptors for CCL3-5 and CXCL9-11, respectively) on CD8+ T cells has emerged as another important regulator of effector T cell recruitment and prognosis in melanoma. For instance, higher expression of CCR5 and CXCR3 was associated with increased T cell infiltration of tumors, lower relapse rates (38) and increased survival in patients with advanced stage III disease (68). In mice CXCR3 deficiency led to faster growth of B16 melanoma (69), while loss of CXCR3 on circulating T cells from patients with melanoma was associated with metastases (70). Notably, CXCR3 signaling appears to be non-redundant and critical for CD8+ T cell trafficking across the endothelium of blood vessels supplying both B16 melanomas and melanoma xenografts (46). Therefore, modulating the CCR5/CCL3-5 and CXCR3/CXCL9-11 chemokine axes can potentially improve CD8+ T cell infiltration of tumors. However, Tregs also express the same receptors and may suppress cytotoxic T cell function.

The role of other chemokines involved in T cell migration into melanoma is less well defined. On the one hand, CXCL12 has been detected in normal and neoplastic tissue (71) and loss of T cells expressing its receptor, CXCR4, was linked to development of lung metastases (70). On the other hand, cytotoxic T cells expressing CXCR4 could migrate into melanoma in response to low concentrations of CXCL12 whereas a high concentration of this chemokine caused T cells to undergo fugetaxis (44, 71–73), highlighting a complex concentration-dependent role for CXCL12 at least in this form of skin cancer.

CCL5 expression in a murine model of spontaneous melanoma correlated with CD3γ expression and T cell recruitment, while increased level of CCR5, the receptor for CCL5, was associated with positive outcomes in melanoma models due to T cell retention in tumors (29). Furthermore, the naturally occurring CCR5Δ32 polymorphism appears to be linked to decreased survival in response to administration of IFN, IL-2, and/or chemotherapy (74). In contrast to these findings, pre-treatment assessment of tumor-infiltrating lymphocytes in patients receiving adoptive T cell therapy showed that patients with the CCR5Δ32 polymorphism displayed an improved response to that therapy (38).

While CD8+ T cell infiltration of solid tumors is generally associated with improved outcomes, other T cell subsets can play dual roles in tumor immunity. For instance, γδ T cells possess both cytotoxic and tumor protective properties (75, 76). CCL2/CCR2 receptor ligand pair has been implicated in γδ T cell recruitment in B16 melanoma where they may exert a cytotoxic effect mediated by IFN-γ, perforin and granzyme B (26). *Mycobacterium bovis* bacillus Calmette-Guérin injection in melanoma patients increased recruitment of γδ T cells *via* CXCL9-11 and CXCR3 (40). Despite this and evidence that γδ T cells are capable of lysing melanoma cells (77), other studies have shown a negative association of increased γδ T cells in circulation in melanoma patients with survival (78). γδ T cells in patients' cutaneous SCC expressed CXCR2-4, CCR2-5, while ligands for these receptors were expressed in cultured SCC supporting a role for these chemokines in γδ T cell recruitment (79).

Another largely overlooked aspect of T cell recruitment is that tumor infiltration is not necessarily a “one-way trip.” Our group has recently demonstrated that effector T cells recruited to solid tumors in the skin can also leave tumors and migrate to tumor draining lymph nodes (80). Thus, the extent of their egress vs. retention may influence the magnitude and nature of the anti-tumor response. Although little is known about the molecular mechanisms guiding these processes, it is likely that chemokines play an important contributory role.

## Systemic Therapy and Immune Cell Infiltration

Systemic therapy can influence the composition of the tumor microenvironment. For instance, chemotherapy causes tumor cell apoptosis, which can in turn induce chemoattractant stress signals. Moreover, therapies such as dacarbazine and temozolomide induce the CD8+ T cell chemoattractants CCL5, CXCL9, and CXCL10 in human melanoma cell lines (29). Consistent with this finding, transcriptomic analysis of 33 cutaneous melanoma metastases resected before or after dacarbazine treatment showed a positive correlation between CCL5, CXCL9 and CXCL10 and CD4, CD8A and CD3Z expression. Thus, patients with high expression of these chemokines after chemotherapy survived longer possibly due to increased T cell recruitment (29). Furthermore, IFN-γ treatment induced chemokines CXCL9 and CXCL10 in melanoma potentially facilitating T cell infiltration (36). Tumors that developed resistance to IFN-γ ceased CXCL9 production and became more tumorigenic (81). Notably, gene expression sequences from melanoma biopsies also revealed a positive correlation between CCL5, CXCL2, CXCL9, and CXCL10 and clinical benefit in a phase II trial of a MAGE-A3 vaccine (39).

Inhibitors of MAPK signaling, such as BRAF and MEK inhibitors, are used to treat BRAF mutated melanoma (82) and can alter the melanoma immune landscape leading to increased T cell infiltration (83–87). When combined with checkpoint inhibitors, BRAF inhibitors enhanced anti-tumor response and improved overall survival (83). These effects may be mediated by altering chemokine-induced immune migration into tumors since BRAF inhibition in melanoma increased serum levels of CCL2, CCL4, and decreased CXCL8 (86), which correlated with increased CD8+ T cell infiltration.

Checkpoint inhibitor therapy in melanoma represents another example where modulation of the chemokine tumor microenvironment occurs as a result of anti-cancer intervention. Administration of anti-PD-1 antibody enhanced migration of the adoptively transferred T cells into B16 melanoma followed by greater tumor regression (88). The tumors had higher expression of IFN-γ and CXCL10 while IFN-γR^−/−^ mice and CXCL10^−/−^ mice showed reduced T cell infiltration. Immunochemical analysis of primary tumor biopsies from patients demonstrated a relationship between higher type 1 IFN expression and higher intratumoral CXCL10 as well as increased numbers of CXCR3^+^ and granzyme B^+^ lymphocytes (89). Furthermore, analysis of gene expression profiles of biopsies collected from 45 melanoma patients before and after commencing anti-CTLA4 (ipilimumab) therapy, showed that in patients with clinical responses to ipilimumab there was increased expression pre- and post-treatment of CXCL9-11, CCL4 and CCL5, chemokines associated with T cell trafficking into melanoma (27). However, some of the above chemokines affect not only T cell recruitment but also innate immune cells.

Recent studies have demonstrated the immense potential of transcriptomics and single-cell RNA-sequencing (RNA-seq) in uncovering chemokine-mediated cellular interactions in melanoma and mechanisms of resistance to immunotherapy (90–93). For instance, transcriptomic studies have identified a checkpoint inhibitor resistance signature, which included monocyte and macrophage chemotactic genes (CCL2, CCL7, CCL8, and CCL13) (90). A recent study uncovered a tumor resistance program that was associated with T cell exclusion and immune evasion, predicted clinical response to checkpoint therapy in melanoma patients. This program could be repressed by cyclin dependent kinase (CDK) 4/6 inhibition suggesting new strategies to negate immunotherapy resistance (91).

## Immune Evasion By Chemokine Augmentation

Immune evasion by skin cancers like melanoma represents one of the mechanisms whereby they proliferate and metastasize (94). Immunosuppressive cells of the innate (discussed above) and adaptive immune systems are recruited to the tumor microenvironment by a range of chemokines. In the case of immunogenic tumors like melanoma, Tregs have the potential to dampen the tumor-specific T cell response. Cutaneous overexpression of CCL22 led to diversion of Tregs from pulmonary melanoma metastases to the skin, leading to inhibition of metastatic growth in the lungs (33). Other studies have shown CCR5-dependent recruitment of Tregs into melanoma and SCC (8, 95), while the ligands for this receptor were produced by MDSCs (8). Consistent with this, CCR5-deficient mice demonstrated slower rate of melanoma growth (8, 95). In another study, B16 melanoma cells overexpressing CCL21 displayed increased growth potential and on injection induced a more suppressive tumor environment containing increased Tregs and TGFβ but decreased IFN-γ levels (9). However, not all retrospective studies of clinical melanoma samples supported the importance of Tregs in modulating anti-tumor responses, indicating the need for more research.

Blocking infiltration of cytotoxic effector cells into tumors is another possible mechanism of evasion and may be one of the reasons why checkpoint inhibitors are not always effective for melanoma treatment (96). Evidence for T cell blockade comes from analysis of a metastatic model of B16 melanoma. In this model, CD8+ T cell infiltration was dependent on the two CXCR3-cognate ligands, CXCL9 and CXCL10, but once metastatic spread had occurred both chemokines were decreased in metastatic lesions (37, 97). Furthermore, CXCL9 and CXCL10 expression in primary melanoma samples from patients was associated with greater CD8+ T cell infiltration whereas biopsies taken from metastatic lesions were lower (97). CXCL9 and CXCL10 expression was decreased by adenosine signaling as metastatic melanoma developed in the lungs indicative of the ability of the tumor to suppress the chemokines responsible for attracting the CD8+ T cells (37). Notably, adoptively transferred CXCR3+ tumor-specific CD8+ T cells were capable of infiltrating metastatic tumors in the lungs (37).

## Concluding Remarks

The clinical importance of chemokine-dependent immune cell recruitment into tumors is illustrated by the most recent advances in cancer immunotherapy, in particular checkpoint inhibitor therapy (88, 98), which has been very effective in about a third of patients with melanoma while the remainder have not responded. It is possible that differential chemokine expression may partially explain this all-or-none effect. Manipulating chemokine levels in primary tumors offers the opportunity for selective control of immune cell recruitment into tumors, thereby increasing the therapeutic efficacy of immunotherapy. However, many variables currently prevent the targeting of chemokines from becoming a realistic therapeutic approach. For example, chemokines play a complex multifunctional role in tumor development, growth and metastasis and their effect can be either pro-tumor or anti-tumor. Cells of the innate and adaptive immune systems with opposing functions in tumor immunity can respond to the same chemokines and be recruited into the tumor. Thus, optimization of immunotherapy will depend on further studies of the chemokine receptor-ligand pairs operating in the tumor microenvironment–likewise for anti-cancer vaccines the efficacy of which relies on recruitment of antigen-specific T cells to tumors.

## Author Contributions

All authors listed have made a substantial, direct and intellectual contribution to the work, and approved it for publication.

### Conflict of Interest Statement

The authors declare that the research was conducted in the absence of any commercial or financial relationships that could be construed as a potential conflict of interest.
